# A Study of the Deformities of the Jaws among the Degenerate Classes of Europe

**Published:** 1898-01

**Authors:** Eugene S. Talbot


### International Dental Journal.

Vol. XIX. January, 1898. No. 1.

### Original Communications.[1]

### A STUDY OF THE DEFORMITIES OF THE JAWS AMONG THE DEGENERATE CLASSES OF EUROPE.

BY EUGENE S. TALBOT, M.D., D.D.S.[2]

The Twelfth International Medical Congress in Moscow, Russia, afforded me a long-sought-for opportunity not merely to visit Russia, but also to cover nearly all the countries of Europe.

In visiting the various cities I made special observations of the degenerates in each of the various institutions for the defective classes. The objective points of interest were the prisons, insane hospitals, schools of idiocy, foundlings' homes, etc. The features of the soldiers, police, and cabmen, as well as the citizens themselves, were incidentally noted for the purpose of comparison. These observations, however, were for an entirely different purpose, the object of this paper being to record results as to the deformities of the jaws and teeth of the mature degenerate classes.

In a prison in Athens containing four hundred and fifty-two convicts not a single V-shaped or saddle arch was found, although

[1] The editor and publishers are not responsible for the views of authors of papers published in this department, nor for any claim to novelty, or otherwise, that may be made by them. No papers will be received for this department that have appeared in any other journal published in the country.

[2] Fellow of the Chicago Academy of Medicine.

slight irregularities of the teeth due to local causes were observed. Arrest of the lower jaw, however, was the rule, which, together with the recession of the forehead, gave to the individual an idiotic appearance. Irregularity, in the relation of the upper to the lower jaw due to excessive and arrest of development was very common. The third molars, upper and lower, were present, but the vault was lower than the average.

In a Greek insane hospital (idiots are here confined with the insane) in Constantinople, of three hundred and thirty-two inmates (equally divided as to sex), only one case of V-shaped arch was noted; the vaults low; upper jaws large and full; but forty-eight per cent. of the lower jaws were arrested; third molars normally developed.

In an Armenian insane hospital (idiots are here confined with the insane) in Constantinople, of two hundred and fifty inmates (one hundred and seventy-five males, seventy-five females), there was one partial V-shaped arch; the third molars normal, and the lower jaw arrested in eighteen per cent. There were many mongoloid faces.

In the Vienna Insane Hospital, of three hundred and twenty-six insane and idiots, there were four partial V-shaped and one saddle-shaped. The third molars were normally developed in three hundred and eleven cases.

In a prison in Moscow, with two thousand convicts (two hundred and forty-seven of which were in the hospital), there were no contracted jaws or irregularities of the teeth. The jaws were very large and vaults low. In the Moscow Reform School there were one hundred and twelve boys, ranging from ten to eighteen years. Three had partial V-shaped arches. No saddle-shaped arches. The jaws, as a rule, were large and broad, with low vaults.

In a Moscow insane hospital, with four hundred patients, of which twelve were idiots, no contracted arches were observed. The jaws were large and broad, with low vaults.

In the Stockholm Insane Hospital, with two hundred and seventy patients, there were six V-shaped arches, twelve partial V-shaped, four semi-V-shaped, twenty-three saddle-shaped, four partial saddle-shaped, eleven excessively developed upper jaws, three excessively developed lower jaws, nine hypertrophy of the alveolar process, forty-two missing third molars, six missing laterals. Deformities of individual teeth numerous. The School of Idiocy, of Stockholm, with one hundred and twenty inmates (eighty boys, forty girls), gave the following results :

BOYS.

| | |
|---|---|
| Normal jaws | 14 |
| V-shaped | 12 |
| Partial V-shaped | 10 |
| Semi-V-shaped | 4 |
| Saddle-shaped | 8 |
| Partial saddle | 1 |
| Semi-saddle | 2 |
| Hypertrophy of the alveolar process | 32 |
| Macrocephalic | 12 |
| Microcephalic | 5 |

GIRLS.

| | |
|---|---|
| Normal jaws | 15 |
| V-shaped | 1 |
| Partial V-shaped | 5 |
| Semi-V-shaped | 5 |
| Saddle-shaped | 8 |
| Semi-saddle | 1 |
| Hypertrophy of the alveolar process | 14 |
| Macrocephalic | 6 |
| Microcephalic | 4 |

One boy, aged thirteen, who was able to take care of himself, had a head thirty-two inches in circumference, one of the largest on record. The prison at Hamburg had eighteen hundred convicts. Large, well-developed jaws were the rule. Asymmetry in development, however, was frequently noticed, as well as other stigmata.

The School of Idiocy at Hamburg had six hundred children (three hundred and ninety-six boys, two hundred and four girls), and gave the following results:

BOYS.

| | |
|---|---|
| Normal jaws | 62 |
| V-shaped | 12 |
| Partial V-shaped | 16 |
| Semi-V-shaped | 8 |
| Saddle | 4 |
| Partial saddle | 3 |
| Semi-saddle | 2 |
| Hypertrophy of the alveolar process | 46 |
| Macrocephalic | 3 |
| Microcephalic | 4 |

GIRLS.

| | |
|---|---|
| Normal jaws | 28 |
| V-shaped | 4 |
| Partial V-shaped | 7 |
| Semi-V-shaped | 3 |
| Saddle | 1 |
| Partial Saddle | 1 |
| Semi-saddle | 3 |
| Hypertrophy of the alveolar process | 25 |
| Macrocephalic | 5 |
| Microcephalic | 2 |

One boy of thirteen had excessive lower jaw, being one and a half inches beyond normal upper. A most remarkable case.

In the Insane Hospital and School of Idiocy at Amsterdam there were thirteen hundred and thirty insane and two hundred and fifty-five idiots. In the insane no contracted arches were found. Vaults low, sixty-seven. Hypertrophy of the alveolar process. No third molars missing.

IDIOTS.

| | |
|---|---|
| Males | 116 |
| V-shaped | 1 |
| Partial V-shaped | 3 |
| Semi-V-shaped | 1 |
| Saddle | 1 |
| Females | 139 |
| V-shaped | 1 |
| Partial V-shaped | 2 |
| Hypertrophy of the alveolar process | 19 |

The vaults low and jaws well developed.

The School of Idiots, Paris, six hundred and sixty-seven inmates (five hundred boys, one hundred and sixty-seven girls), gave the following results :

BOYS.

| | |
|---|---|
| V-shaped | 1 |
| Partial V-shaped | 40 |
| Semi-V-shaped | 2 |
| Saddle | 2 |
| Partial saddle | 1 |
| Semi-saddle | 4 |
| Hypertrophy of the alveolar process | 7 |

GIRLS.

| | |
|---|---|
| V-shaped | 1 |
| Partial V-shaped | 6 |
| Semi-V-shaped | 1 |
| Saddle | 8 |
| Partial saddle | 2 |
| Semi-saddle | 1 |
| Hypertrophy of the alveolar process | 4 |

The vaults were low.

Having permits to visit the following prisons in Paris, Ci-apés, Le Dépot, Grande Roquette, Mazas, La Santé, St. Pélagie, and St. Lazan, after examining the convicts in the first four, aggregating two thousand six hundred, I abandoned the task, since no deformities of the jaws were observed of special value.

In England I examined the following public asylums: 1. Earlswood Idiot Asylum. 2. Darenth School for Children. 3. Darenth School for Adults, Hanwell Hospital for the Insane, and the following private institutions: 4. Mrs. Langdon Down. 5. Dr. Shuttleworth. 6. Dr. Beach.

A day was spent at the Criminal Insane Hospital, Broadmore. Fully one-half the inmates were so violent that the task was given up. Enough, however, was observed to warrant my stating that fully eighty to eighty-five per cent. had marked deformities of the jaws and teeth.

Hanwell Hospital for the Insane, Southall, had two thousand and eighty patients. These people were mostly of the dependent class, who went insane after maturity; hence the class of deformities which are under discussion were not common. Hypertrophy of the alveolar process and excessive and arrested development of the jaws were, however, frequently noticed. Stigmata of degeneracy of head, face, eyes, ears, body, hands, and feet were the rule.

Earlswood Idiot Asylum, Red Hill, Surrey, contained six hundred and seventy, of which four hundred were boys and two hundred and seventy girls.

BOYS.

| | |
|---|---|
| Normal jaws | 31 |
| V-shaped | 108 |
| Partial V-shaped | 69 |
| Semi-V-shaped | 11 |
| Saddle | 19 |
| Partial saddle | 27 |
| Semi saddle | 13 |
| Marked arrest of upper jaw | 104 |

| | |
|---|---|
| Marked protrusion of upper jaw | 64 |
| Marked protrusion of lower jaw | 11 |
| Marked arrest of lower jaw | 306 |
| Lateral incisors arrested | 46 |
| Lateral incisors lost | 28 |
| Third molars lost | 180 |
| Showed malnutrition of teeth | 160 |

GIRLS.

| | |
|---|---|
| Normal jaws | 24 |
| V-shaped | 67 |
| Partial V-shaped | 86 |
| Semi-V-shaped | 24 |
| Partial saddle | 8 |
| Semi-saddle | 23 |
| Cleft palate | 1 |
| Marked arrest of upper jaw | 87 |
| Marked protrusion of upper jaw | 24 |
| Marked protrusion of lower jaw | 1 |
| Marked arrest of lower jaw | 237 |
| Lateral incisors arrested | 30 |
| Lateral incisors lost | 16 |
| Third molars lost | 85 |
| Showed malnutrition of teeth | 78 |

Darenth School for Idiots, Dartford, Kent, had one thousand inmates (six hundred and forty boys, three hundred and sixty girls).

BOYS.

| | |
|---|---|
| Normal jaws | 150 |
| V-shaped | 143 |
| Partial V-shaped | 140 |
| Semi-V-shaped | 105 |
| Saddle | 35 |
| Partial saddle | 20 |
| Semi-saddle | 10 |
| Marked arrest of upper jaw | 450 |
| Marked protrusion of upper jaw | 150 |
| Marked protrusion of lower jaw | 23 |
| Arrest of lower jaw | 600 |
| Lateral incisors arrested | 68 |
| Lateral incisors lost | 42 |
| Third molars lost | 388 |
| Hypertrophy of upper jaw | 150 |

GIRLS.

| | |
|---|---|
| Normal jaws | 90 |
| V-shaped | 118 |
| Partial V-shaped | 80 |

| | |
|---|---|
| Semi-V-shaped | 65 |
| Partial saddle | 8 |
| Semi-saddle | 20 |
| Marked arrest of upper jaw | 310 |
| Marked protrusion of upper jaw | 90 |
| Marked protrusion of lower jaw | 9 |
| Arrest of lower jaw | 340 |
| Lateral incisors arrested | 32 |
| Lateral incisors lost | 19 |
| Third molars lost | 111 |
| Hypertrophy of upper jaw | 90 |

Darenth School for Adults (Idiots), Dartford, Kent, contained one thousand and fifty inmates (four hundred and fifty males, six hundred females).

MALES.

| | |
|---|---|
| Normal jaws | 60 |
| V-shaped | 105 |
| Partial V-shaped | 93 |
| Semi-V-shaped | 53 |
| Saddle | 31 |
| Partial saddle | 5 |
| Marked arrest of upper jaw | 295 |
| Marked protrusion of upper jaw | 162 |
| Marked protrusion of lower jaw | 8 |
| Arrest of lower jaw | 409 |
| Lateral incisors arrested | 48 |
| Lateral incisors lost | 37 |
| Third molars lost | 442 |
| Hypertrophy of upper jaw | 58 |

FEMALES.

| | |
|---|---|
| Normal jaws | 40 |
| V-shaped | 177 |
| Partial V-shaped | 121 |
| Semi-V-shaped | 79 |
| Partial saddle | 8 |
| Semi-saddle | 10 |
| Marked arrest of upper jaw | 436 |
| Marked protrusion of upper jaw | 209 |
| Marked protrusion of lower jaw | 17 |
| Arrest of lower jaw | 580 |
| Lateral incisors arrested | 72 |
| Lateral incisors lost | 62 |
| Third molars lost | 597 |
| Hypertrophy of upper jaw | 36 |

Of the children, five hundred and seventy-six boys showed malnutrition in utero; two hundred and eighty-two girls showed malnutrition in utero. Of the adults, three hundred and ninety-six males showed malnutrition in utero; five hundred and seventy-eight females showed malnutrition in utero.

Mrs. Langdon Down's School for Idiots, Normansfield, Hamptonwick, contained one hundred and forty-seven inmates (ninety-seven boys, fifty girls).

BOYS.

| | |
|---|---|
| Normal jaws | 12 |
| V-shaped | 36 |
| Partial V-shaped | 20 |
| Semi-V-shaped | 15 |
| Saddle | 9 |
| Partial saddle | 13 |
| Semi-saddle | 28 |
| Arrest of upper jaw | 86 |
| Third molar missing | 92 |
| Lateral incisors missing | 16 |
| Teeth showing arrest and grooves | 46 |
| Hypertrophy of the alveolar process | 19 |

GIRLS.

| | |
|---|---|
| Normal jaws | 5 |
| V-shaped | 10 |
| Partial V-shaped | 9 |
| Semi-V-shaped | 12 |
| Saddle | 7 |
| Partial saddle | 1 |
| Semi-saddle | 16 |
| Arrest upper jaw | 45 |
| Third molar missing | 47 |
| Lateral incisors missing | 8 |
| Teeth showing arrest and grooves | 21 |
| Hypertrophy of the alveolar process | 7 |

Of the twelve normal dental arches (males) seven were hypertrophied. Of the five normal dental arches (females) three were hypertrophied.

Dr. Shuttleworth, Private Idiot School, Richmond Hill, had twelve boys and girls. There was one normal jaw, but no laterals. There were two V-shaped, five partial V-shaped, one semi-V-shaped, two partial saddle, and one semi-saddle-shaped jaw. Four hypertrophy of alveolar process. Nine had notched and pitted teeth, and all high vaults. These patients were too young to decide as to the number of third molars, but five had one or both laterals missing.

Dr. Fletcher Beach, Winchester House, Kingston Road, had thirteen patients. There were three V-shaped, eight partial V-

shaped, and one semi-V-shaped jaw; six hypertrophy of the alveolar process; eight had notched and pitted teeth; all high vaults. These patients were also too young to decide as to number of third molars. Four had one or both laterals missing.

These reports are tabulated in the order in which they were made. They show a gradual increase of degeneracy from the examinations made in Greece to those in England. It will also be noticed that the deformities of the jaws and teeth are more numerous among the better classes, such as are shown in the private institutions of Mrs. Langdon Down and Drs. Shuttleworth and Beach, than among those of the poorer classes in the public institutions of England.

From examinations previously made in Spain, Italy, and Switzerland among the degenerate classes, a very small percentage of deformities of the teeth and jaws was found. As compared with the American-born degenerate classes, the percentages are greater than those of the Latin races, and the Slavs, Germans, Austrians, Danes, and Dutch, but from twenty-five to thirty-five per cent. less than the Swedes and English.

These observations have proved to me what I long ago suspected from my studies of the degenerate classes which have come to America, and which fill our public charitable institutions as well as our prisons, that the higher the intellectuality the greater the degeneracy of the jaws and teeth.

